# Rethinking viral vector quantification: a microfluidic approach to standardised functional titre assays

**DOI:** 10.3389/fbioe.2026.1720882

**Published:** 2026-04-13

**Authors:** Daria Andreea Farcas, Charles Moore-Kelly, Philippa Stevenson, Rui André Saraiva Raposo, Marco P. C. Marques, Nicolas Szita

**Affiliations:** 1 Department of Biochemical Engineering, University College London, London, United Kingdom; 2 Oxford Biomedica (UK) Limited, Oxford, United Kingdom

**Keywords:** analytics, CAR-T cells, cell and gene therapies (CGT), functional titre, lentiviral vectors, microfluidics, multiplicity of infection (MOI), transduction

## Abstract

The quantification of lentiviral vector (LVV) potency via titration is a critical quality control step for cell and gene therapies. However, standard functional titre assays are fundamentally limited by an inability to detect multiple integration events, procedural variations, and, most critically, a mass transport limitation created by the fluid overlay in conventional well plates. These issues, compounded by a lack of standardisation, lead to significant inter-laboratory variability and a systematic underestimation of true vector potency. In this study, we employed a microfluidic approach to create a more precisely engineered assay environment. We systematically evaluated channel depth, incubation time, vector concentration, and multiplicity of infection (MOI) for their impact on assay linearity, sensitivity, limit of detection, and reproducibility. A 0.2 mm deep channel provided linearity and reproducibility comparable to 96-well plates yet offered shorter incubation periods, and enhanced sensitivity, detecting activity down to a MOI of 0.0625 (corroborated by qPCR analysis) - a level at which conventional well plates fail. This establishes our microfluidic platform as a device-based analytical standard that transforms functional titre quantification from a variable protocol into a more reliable engineering solution for quality testing in cell therapy manufacturing.

## 1 Introduction

Cell and gene therapies (CGTs) have emerged as promising approaches for treating various neoplastic disorders, with Chimeric Antigen Receptor T (CAR-T) cells showing particularly strong efficacy in haematological malignancies such as Acute Lymphoblastic Leukaemia (ALL) (Bezeljak, 2022). These immunotherapies rely on the stable *ex vivo* modification of human T cells to express a CAR on their surface, enabling them to recognise a tumour-specific antigen (Poorebrahim et al., 2020). Among the different delivery methods, lentiviral vectors (LVVs) are one of the most suitable due to their ability to stably integrate transgenes into both dividing and non-dividing cells (Naldini et al., 1996). As of 2022, LVV vectors accounted for up to 66% of all *ex vivo* gene modifications employed in UK clinical trials (CGT Catapult, 2022). LVV demand is projected to increase significantly with 50–75 CGTs expected to be approved in the US alone by 2030 (Choe et al., 2022). Moreover, recent reports have documented the use of LVV expanding beyond the CAR-T cell therapy field, with considerable evidence currently supporting their potential efficacy in the treatment of solid tumours or autoimmune diseases (Du et al., 2025; Yin et al., 2025). The success of these therapies is fundamentally dependent on the consistent production of high-quality vectors, necessitating robust analytical methods to define, monitor, and ensure their quality.

Analytical assays typically evaluate identity, potency, safety and purity of a lentiviral sample (Shi et al., 2022). A particularly relevant measure of potency is the functional titre, which quantifies the number of lentiviral particles capable of integrating the gene of interest and driving transgene expression in target cells *in vitro.* Functional titre directly determines the vector dose required for successful CAR-T transduction, which often necessitates Multiplicity of Infection (MOI) values resulting in less than 5 vector copies per cell (Wagner et al., 2022). Recent functional titres are reported at approximately 2 × 10^7^ TU mL^-1^ for transiently transfected systems (Williams-Fegredo et al., 2024a) and 2 × 10^8^ TU mL^-1^ (Broussau et al., 2023; Tridgett et al., 2024) for stable producer cell lines. Accurate, reproducible measurement of this parameter is therefore essential to ensure the quality of LVV products.

The standard method for quantifying LVV functional titre is a cell-based flow cytometry assay typically performed using HEK293 cells. Serial dilutions of the LVV sample are incubated with a known quantity of target cells. Following the expression of the protein of interest, which may require immunostaining if the transgene does not express a reporter protein, the fraction of positive cells is quantified by flow cytometry (Perry and Rayat, 2021). A key limitation of this assay is its inability to detect multiple integration events within a single cell, which can lead to an underestimation of the (true) functional titre. To address this underestimation issue, functional titre calculations are typically based on a transduction efficiency of ≤ 30%, assuming a Poisson distribution of infections (Shabram and Aguilar-Cordova, 2000). Additionally, the total assay timeframe varies, with reports ranging from 72 h (Williams-Fegredo et al., 2024b) to approximately 120 h (Labisch et al., 2021). This timeframe also includes an extended incubation of the LVV particles with the cells, widely ranging from 4 h (Geraerts et al., 2006) to 18 h (Labisch et al., 2021) or 72 h (Williams-Fegredo et al., 2024a) in the instance when fresh media is not supplemented to the cells post-transduction.

Fundamentally, the assay is also compromised by physical constraints understood for decades. Seminal work established that LVVs are only able to diffuse an effective distance of ∼600 µm before their half-life at 37 °C leads to degradation (Chuck and Palsson, 1996; Higashikawa and Chang, 2001). This physical reality is in direct conflict with the geometry of the standard assay environment: a microwell plate with a culture fluid overlay of 1–3 mm. The thickness of this overlay forces most viral particles to traverse distances where they are likely to decay before reaching a target cell. This challenge was long recognised in classical virology, where it was standard practice to “lay as thin a layer of a viral preparation as possible over the target cells for as long as practical” to minimise titre underestimation (Shabram and Aguilar-Cordova, 2000), with data suggesting layers in the tenths of a millimetre (Dulbecco and Vogt, 1954). When combined with the other issues, such as the inability to detect multiple integration events and variation in procedural standards, this results in a field where titres from different labs become difficult to compare, presenting a significant challenge to the development and quality control of CGT products (Comisel et al., 2021; McCarron et al., 2016; Merten et al., 2016; Sinn et al., 2005).

Microfluidics offers an approach to re-engineer the assay environment by precisely controlling the cellular microenvironment (Aranda Hernandez et al., 2022; Asadian et al., 2024; Gouvarchin Ghaleh et al., 2020), minimising the diffusion path length between vector and cell to a scale where transport is no longer limiting. This principle has been previously employed to enhance LVV gene transfer kinetics (Tran et al., 2017), to explore cost-effective scalability for manufacturing (Moore et al., 2019), and to intensify transduction of hard-to-transduce cells (Ahmadi et al., 2023). Critically, Tran et al. (2017) reported vector “utilisation efficiencies” greater than 100% in microchannels, a paradox that directly implicates that mass transport limitations in conventional well plates relate to the underestimation of vector potency. We hypothesised this paradox of “greater than 100% efficiency” points to an opportunity for analytics, where a microfluidic environment, which reveals the hidden potency masked by the diffusion limitations of standard plates, could be leveraged to create a more sensitive and accurate functional titre assay. In this contribution, we therefore systematically evaluate channel depth, viral vector concentration, and MOI, and rigorously analyse assay linearity, sensitivity, limit of detection, and reproducibility. Furthermore, we compare our flow cytometry results with qPCR to further validate a microfluidics-based analytical assay. Our results demonstrate that a microfluidic approach can create a functional titre assay that is not only more sensitive and economical but also more reproducible and standardised.

## 2 Materials and methods

### 2.1 Routine cell culture maintenance

Human Embryonic Kidney cells (HEK293T) were obtained from the UCL tissue culture facility and cultured in Dulbecco’s Modified Eagle Medium (DMEM) (Thermo Fisher Scientific, United Kingdom) supplemented with 10% Foetal Bovine Serum (FBS), 1% Penicillin/Streptomycin, and 1% GlutaMAX (Thermo Fisher Scientific, United Kingdom). Routine passaging was performed through the addition of trypsin-EDTA 0.25% solution (Thermo Fisher Scientific, United Kingdom). This media composition was used across all experiments involving HEK293T cell culture unless otherwise stated. Further cell line authentication was not performed.

### 2.2 Transient transfection using the third-generation lentiviral plasmid system

HEK293T cells were seeded in a T175 CellBind flask (Corning, United Kingdom) at 7.5 × 10^4^ cells cm^-2^, and incubated for 16 h at 37 °C and 5% CO_2_. A proportion of 1.5 µg plasmid DNA per 1.0 ×10^6^ cells seeded was used for transfection, summing up a total of 19.5 µg using an equal ratio of the 4 plasmids comprising a third-generation lentiviral plasmid system: pRSV-Rev, pLJM1-EGFP, pMDLg/pRRE, pMD2.G. The plasmids were a gift from Didier Trono (Addgene plasmids #12253, #19319, #12251, #12259) (Dull et al., 1998). A transfection solution was made with a 1:1 volumetric ratio between PEIpro (Polyplus, United Kingdom) and the mixture of plasmid DNA diluted in OptiMEM (Thermo Fisher Scientific, United Kingdom). The transfection solution is added to the flask and incubated for 24 h at 37 °C and 5% CO_2_. A media change was performed after 24 h, using complete media supplemented with 5 mM sodium butyrate (Cambridge Bioscience, United Kingdom). The media collected at 48 h post-transfection was filtered through a 0.45 µm polyether sulfone (PES) syringe filter (Merck, Germany) and concentrated using the Lenti-X Concentrator reagent (Takara Bio, France) according to the manufacturer’s instructions. The LVV material was aliquoted and stored at −80 °C.

### 2.3 Functional titre quantification in 96-well plates

HEK293T cells were seeded in a flat-bottom 96-well plate (Thermo Fisher Scientific, United Kingdom) at 7.5 × 10^4^ cells cm^-2^ and incubated for 24 h at 37 °C and 5% CO_2_. Each lentiviral dilution and a non-transfected control were measured in triplicates. After 24 h, the cell count was determined by trypsinization and quantification on the NucleoCounter NC-3000 (ChemoMetec, Denmark). The plate was then incubated with 50 µL lentiviral sample for 48 h at 37 °C and 5% CO_2_. The cells were harvested, fixed using 4% paraformaldehyde in Phosphate Buffered Saline (PBS) (Thermo Fisher Scientific, United Kingdom) and run on a LSRFortessa™ flow cytometer (BD Biosciences, United Kingdom) with a 10,000 events limit. The data was analysed using FlowJo v10.8.1, and the functional titre of the lentiviral vector material was calculated using Equation 1.

### 2.4 Polydimethylsiloxane (PDMS) casting and characterisation by optical profilometry

Microchannel moulds were designed in Fusion v2.0.20981 (Autodesk, United States), manufactured using CNC milling from 3 mm thick polycarbonate (PC) sheets. PDMS was cast into the PC mould using a SYLGARD™ 184 Silicone Elastomer Kit (Dow, United States) 10:1 w/w ratio of elastomer to curing agent preparation. The moulds were degassed for 30 min in a vacuum chamber, cured at 80 °C for 2 h, and then cut out using a scalpel. The channel depths (PC mould and corresponding cast PDMS layer) were measured with a 3D optical profilometer Contour GT-100 using a 5× objective and analysed in the Vision64 v5.41 software (Bruker, United States) through the Step Height function.

### 2.5 Chemical bonding of cyclo-olefin copolymer (COC) and PDMS layers

A 1 mm thick COC microscope slide (Microfluidic ChipShop, Germany) was treated by a corona plasma treater (Elveflow, France) for 2 min, followed by incubation in a 2% (3-Aminopropyl)-triethoxysilane (APTES) (Merck, Germany) in PBS (Thermo Fisher Scientific, United Kingdom) solution for 20 min at room temperature. The slide was then washed in distilled water (Thermo Fisher Scientific, United Kingdom) and dried by air stream. The surface modified microscope slide and the PDMS layer forming the microfluidic channels were treated by a corona plasma treater (Elveflow, France) for 2 min before being manually placed in contact and incubated at 80 °C for 16 h to reinforce the bonding.

### 2.6 Validation of device sterility

All microchannels were submerged in distilled water (Thermo Fisher Scientific, United Kingdom) and sterilised by autoclaving at 121 °C for 20 min. The PDMS and COC parts were separated and incubated overnight at 37 °C in LB Broth (Thermo Fisher Scientific, United Kingdom) alongside non-sterilised counterparts and a negative control. The supernatant was then analysed by spectrophotometry at 600 nm to verify the extent of bacterial growth in each culture using a Jenway UV/Visible Single Beam Spectrophotometer (Fisher Scientific, United Kingdom). An additional sterility validation experiment was performed by incubating the PDMS and COC parts for 14 days at 35 °C in Fluid Thioglycolate Medium (FTM) (Fisher Scientific, United Kingdom) according to the United States Pharmacopeia (USP) guidelines. The results were evaluated through visual observation of the culture media.

### 2.7 Routine cell culture maintenance and lentiviral transduction in microchannel format

HEK293T cells were seeded at a density of 7.5 × 10^4^ cells cm^-2^ and media changes performed every 24 h. The cell viability and total cell count were obtained by harvesting cells at specified time intervals, and quantified using the Viability and Count protocol on the Nucleocounter NC-3000 (ChemoMetec, Denmark). Microscopy was performed using an EVOS M5000 microscope (Thermo Fisher Scientific, United Kingdom) for cell morphology observation. Lentiviral vector material diluted in complete media was added at 24 h post cell seeding at the concentration corresponding to the specified MOI. Media changes are performed at 24 h post-transduction, unless otherwise stated. The cells are then harvested at 48 h post-transduction and analysed via flow cytometry using the procedure stated in 2.3. To assess assay reproducibility, the transduction efficiency obtained across separate experimental occasions was analysed using the coefficient of variation (CV) (Equation 2). For linearity evaluation, the transduction efficiency data was normalised using Equation 3.

### 2.8 Quantification of vector genome integration

Cells from microchannels or from 96-well plates were collected by centrifugation at 300 RCF for 5 min, washed once in sterile PBS, and the cell pellets stored at −80 °C until further sample processing. Genomic DNA was isolated from cell pellets according to an automated protocol, using a QIAamp 96 DNA kit (Qiagen, Germany) with a QIAcube HT (Qiagen, Germany). The elution was then stored at −20 °C until further processing. The number of vector copies per cell was identified by quantifying the vector packaging signal (Ψ) in extracted DNA samples and normalising this value to the number of copies of the housekeeping gene, ribonuclease P RNA component H1 (RPPH1), via duplex qPCR. Both sequences were quantified by interpolation from a corresponding 5-points DNA standard curve with known number of target copies. The 96-well qPCR plates (Thermo Fisher Scientific, United Kingdom) were prepared by combining the PCR Master Mix (Thermo Fisher Scientific, United Kingdom) with genomic DNA and corresponding primers as well as generating the standard curves using a Hamilton STAR Automated Liquid Handler (Hamilton, United States). Corresponding oligonucleotides were added for each target sequence, the HIV-1 Ψ and RPPH1 copies being quantified using specific primers, showed in Table 1. Data was generated and analysed using a QuantStudio 7 Pro Real-Time PCR System (Thermo Fisher Scientific, United Kingdom). Assay controls included a non-template control (NTC) to ensure the lack of template DNA contamination during the extraction procedure.

**TABLE 1:** Primer and probe sequences used for qPCR against the HIV-1 Ψ and RPPH1 targets.

Target	Sequence type	Sequence
A region of the integrated HIV-1 packaging signal (Ψ)	Forward primer	5′ TGGGCAAGCAGGGAGCTA 3′
	Reverse primer	5′ TCC​TGT​CTG​AAG​GGA​TGG​TTG​T 3′
	Probe	5′ FAM-AACGATTCGCAGTTAATCCTGGCCTGTT-TAMRA 3′
RPPH1	Forward primer	5′ CCC​TAG​TCT​CAG​ACC​TTC​CCA​AG 3′
	Reverse primer	5′ GCG​GAG​GGA​AGC​TCA​TCA​G 3′
	Probe	5′ VIC-CCACGAGCTGAGTGCGTCCTGTCATAMRA 3′

### 2.9 Statistical analysis

All data is presented as individual data points with their mean ± one standard deviation. All statistical analysis was performed using GraphPad Prism v8.4.2 (GraphPad, United States). According to the experiment, either a paired or un-paired T-test was used or a one-way ANOVA with either Dunnett’s or Tukey’s *post hoc* analysis with a 95% confidence interval and statistical significance reported as *: p < 0.05; **: p < 0.01; ***: p < 0.001; ****: p < 0.0001.

### 2.10 Equations

$$\text{Functional titre }\left(\text{TU }{\text{mL}}^{‐1}\right)=\frac{N_{cells}\times P\times D}{V\;\left(mL\right)\times 100}$$ (1)

Formula for calculating the functional titre, where N_cells_ is total number of cells, P is the percentage of GFP positive cells, V is the sample volume, D is the LVV dilution factor (Equation 1).

$$\text{CV}=\frac{SD}{Mean}\;x\;100$$ (2)

Formula for calculating the coefficient of variation (CV %), where SD is the standard deviation and the mean is the average of triplicate measurements (Equation 2).

$$\text{Normalized transduction efficiency}=‐\mathrm{Ln}\left(1\;‐\;\frac{P}{100}\right)$$ (3)

Normalisation of transduction efficiency using the Poisson distribution, where P is the percentage of GFP positive cells (Equation 3).

## 3 Results

The observation of “greater than 100% utilization efficiency” in microfluidic transduction systems (Tran et al., 2017) not only suggested that standard titration assays are failing to capture the full potency of lentiviral vectors but also pointed to a clear opportunity to use microfluidics to develop a more insightful analytical tool. We therefore explored whether a microfluidic environment, which minimises the diffusion path between vector and target cell, could be engineered to create a more sensitive and accurate–and ultimately more standardised - functional titre assay. To test this, we systematically characterized the impact of channel depth, incubation time, and vector concentration on key assay performance metrics.

### 3.1 Microchannel fabrication and characterization by optical profilometry

**FIGURE 1:**
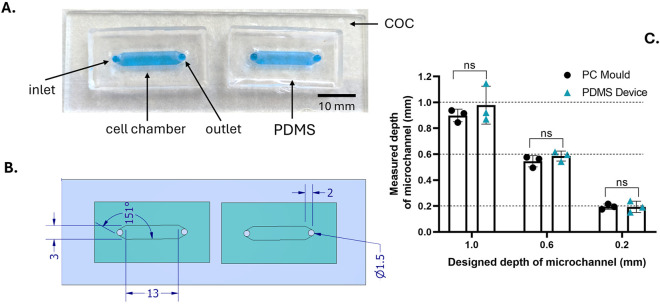
Design and physical characterization of the microchannel device. **(A)** Photographic image of two microfluidic devices. Each device features one inlet and one outlet connector to a cell culture chamber (filled with methylene blue dye for better visualisation). The microchannel structures were made from polydimethylsiloxane (PDMS) and bonded to a cyclo-olefin copolymer (COC) microscope slide. Scale bar = 10 mm. **(B)** Top view drawing of the microchannel device with corresponding key dimensions represented in millimetres. The area of the culture chamber is approximately 0.39 cm^2^, similar to the surface area of a single well of a 96-well plate (0.32 cm^2^). **(C)** Characterisation of microchannel depth by optical profilometry. The micro-machined height of the polycarbonate (PC) mould and the corresponding depths of the PDMS channels were compared to verify the accuracy of the PDMS casting. The channel depths expected by the design are indicated by the dashed lines. All data points are presented as mean ±1 standard deviation of technical triplicates. Significance was assessed using un-paired T-test (p > 0.05).

A set of microfluidic channels of 0.2-, 0.6- and 1.0-mm depth was manufactured using polydimethylsiloxane (PDMS) casting and chemical annealing onto a cyclo-olefin co-polymer (COC) substrate (Figure 1A). To allow for a direct comparison in the case of an adherent cell line transduction, the surface area of the microchannels was designed to be similar to a 96-well plate well, measuring approximately 0.32 cm^2^ (Thermo Fisher Scientific, 2006). The enclosed structures of microfluidic channels offered reduced fluid volumes (relative to 96-well plates), which helped to mitigate media evaporation over long incubation periods at 37 °C. Consequently, the operating volumes corresponding to the 3 channel sizes were approximately 42, 25 and 8 μL, as derived from the dimensions of the Computer Aided Design (CAD) design. The minimum channel depth of 0.2 mm was selected to facilitate accurate culture operations through pipetting while minimizing the impact of media evaporation on the culture characteristics. Smaller channel depths were avoided due to the increased pressure required for media displacement and propensity for channel deformation or collapse during operation.

Polydimethylsiloxane (PDMS) is a commonly used substrate within microfluidic cell culture applications, with desirable properties such optical transparency and high capacity for gas exchange (Torino et al., 2018). A chemical surface modification approach was employed to seal the PDMS structure onto a COC microscope slide. This modification involved air plasma activation followed by surface treatment using APTES solution. Similar approaches have been previously reported (Agha et al., 2024; Cortese et al., 2011), although they have not been validated for cell culture applications. Due to the nature of the manually operated application, the system was expected to be under negligible pressure. The efficiency of the chemical bonding process was confirmed through visual observation and the absence of leakage during the typical fluidic operation and incubation over approximately 96-h. In addition, in the absence of APTES treatment, HEK293T cells have the capacity to attach and grow on both untreated COC and COC treated with plasma (Harvey, 2023).

Overall, the assembly was compatible with monitoring through phase contrast and fluorescence microscopy due to its favourable optical transparency, low autofluorescence and conventional dimensions of a microscope slide (Agha et al., 2022). In addition, COC was identified as suitable substrate due to its biocompatibility (Bernard et al., 2017) and capacity to support the adherence and maintenance of HEK293T cells in culture (Kneitz et al., 2022). Furthermore, both substrates can withstand autoclaving conditions (steam at 15 psi, 121 °C, 20 min), therefore being suitable for cell-based applications. A simple architecture enabled a cell culture region placed between the 2 inlet ports, as detailed in Figure 1B. The aim of this device architecture was to provide a simple method for probing the reliance of transduction efficiency upon a series of cell culture fluid overlays. In addition, this design facilitated high-throughput device production, resulting in single-use cell culture structures. This approach allowed for generating microchannels of various depths in an inexpensive, rapid and high-throughput manner, relying on a polycarbonate (PC) mould design obtained through Computer Numerical Control (CNC) milling. The PC mould as well as the corresponding PDMS channels were characterized using optical profilometry to evaluate the channel depth obtained for each of the 3 selected instances.

Figure 1C compares the depths of the PC mould and PDMS channels with the expected depth according to the CAD. As expected, the results indicated no statistically significant difference (p > 0.05, unpaired t-test) between the measured depths of the PC mould and the corresponding PDMS channels for all three depths tested (Miranda et al., 2022).

### 3.2 Validation of device sterility

**FIGURE 2:**
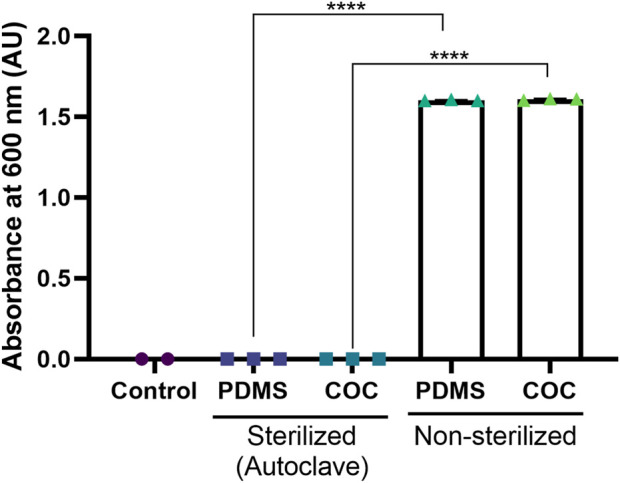
Validation of device sterility. COC and PDMS device parts were incubated overnight at 37 °C in LB medium. The bioburden was quantified by measuring the absorbance of the supernatant at 600 nm before and after sterilisation by autoclaving at 121 °C for 20 min, relative to a negative control. All data is expressed as mean ±1 standard deviation of triplicate absorbance measurements from one experiment. Significance was assessed using un-paired T-test (p < 0.0001).

Microchannel sterility prior to cell seeding is essential for successful cell culture experiments. PDMS and COC were the two materials comprising the microchannel and were therefore in direct contact with the cell culture. Therefore, to evaluate the suitability of autoclaving and validate the sterility of the device, we first compared the levels of bioburden present on both autoclaved and unsterilised device parts. The results demonstrated that both substrates were sterile after one autoclave cycle (Figure 2). Negative (LB media incubated in the absence of any device parts) and positive (LB media containing device parts which have not been autoclaved) control experiments supported this conclusion. The differences between the autoclaved and non-sterilized parts was statistically significant (p < 0.0001, one-way ANOVA) To further validate device sterility, we incubated the device parts for 14 days at 35 °C in Fluid Thioglycolate Medium (FTM) according to US Pharmacopeia (USP) guidance. Visual observation confirmed no growth in the cultures (Supplementary Figure S1).

### 3.3 Lentiviral transduction performed in microchannel format

**FIGURE 3:**
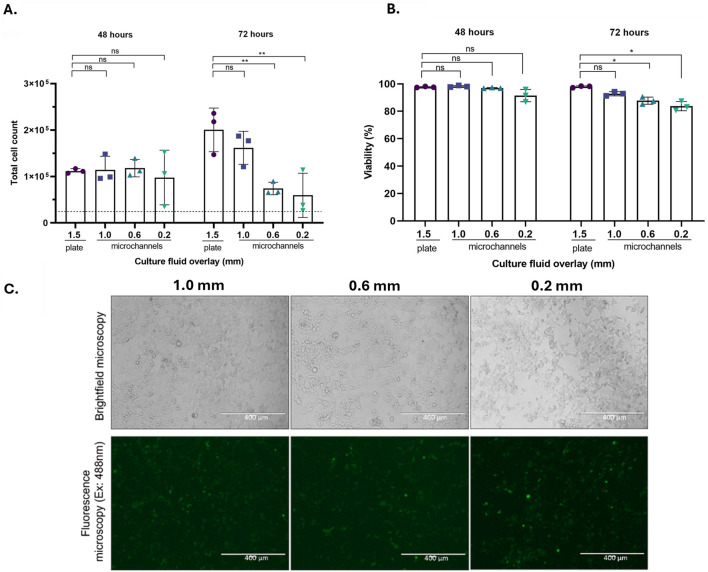
Comparative cell culture analysis of microchannels (depths of 1 mm, 0.6 mm and 0.2 mm) with a 96-well plate. **(A)** Total cell count 48 and 72 h after seeding–dashed line represents the number of seeded cells. **(B)** Cell viability (%) 48 and 72 h after seeding. **(C)** Representative microscopy images (×10 magnification) of cell culture and transduction within the microchannels. All images were acquired at 48 h after transduction, performed at a constant MOI of 2. Cell confluency was monitored by phase contrast microscopy, GFP expression identified by fluorescence microscopy (excitation wavelength of 488 nm). Scale bar = 400 µm. Data points are presented as mean ±1 standard deviation of triplicate wells or channels. Significance was assessed using one-way ANOVA (ns: p > 0.05; *: p < 0.05; **: p < 0.01).

Initial cell culture experiments were conducted to assess the suitability of the microchannels with various culture fluid overlays for maintaining HEK293T cells over 72 h. All 3 channel depths kept both total cell count (Figure 3A) and viability (Figure 3B) to a level comparable to 96-well plates for up to 48 h (p > 0.05, one-way ANOVA, Tukey’s *post hoc* analysis). No detrimental effects were observed on the cell morphology, suggesting suitability for routine cell maintenance and cell-based assays (Figure 3C).

Accurate functional titre quantification requires a consistent cell count at the time of viral addition. We confirmed no statistically significant difference for 48 h after cell seeding (Figure 3A, p > 0.05, one-way ANOVA, Tukey’s *post hoc* analysis). In addition, there was no discrepancy in cell viability when the 1 mm microchannel was compared with the plate (p > 0.05, one-way ANOVA, Tukey’s *post hoc* analysis. However, there was a statistically significant decrease in cell viability and count when comparing the 0.2 mm microchannel to the 96-well plate well at 72 h after cell seeding (p < 0.05 and p < 0.01, one-way ANOVA, Tukey’s *post hoc* analysis). This might point an insufficient rate of media exchange, which was performed manually using pipettes. The decrease was more pronounced for the shallower microchannels. However, since cells are harvested for flow cytometry 72 h post-seeding in a typical assay protocol, the microchannels were considered suitable for performing the cell-based functional quantification assay up until this time-point.

Due to the varying volumes (42, 25 and 8 µL) in the microchannels, adding the same number of viral vector particles would result in different LVV concentrations; smaller culture fluid overlays, for example, would yield higher LVV concentration. Therefore, when conducting lentiviral transductions in microchannels of different depths (hence, volumes), we either kept the multiplicity of infection (MOI) or the LVV concentration constant.

**FIGURE 4:**
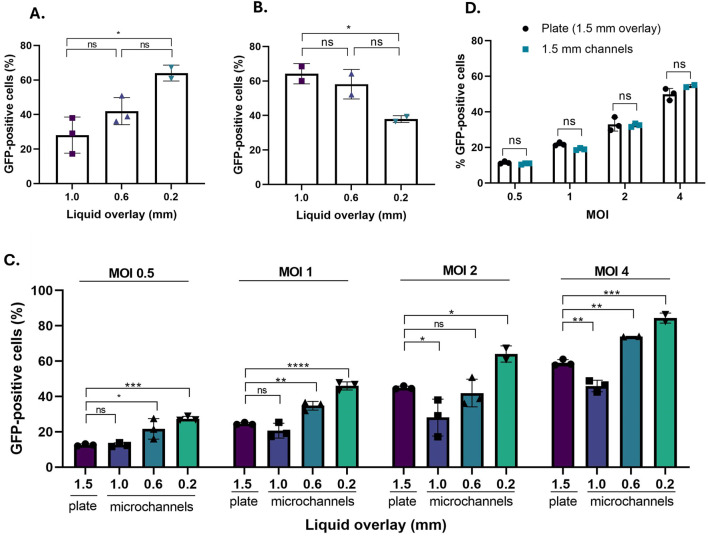
Comparison of transduction efficiency (TE) obtained by maintaining constant either the **(A)** MOI of 2 or the **(B)** LVV concentration (TU mL^-1^) of 6.3 × 10^5^ TU mL^-1^. This was evaluated across 3 microchannels with different culture fluid overlay with a 24h incubation between the LVV particles and target cells. **(C)** TE evaluated across a range of MOI values: 0.5, 1, 2 and 4. All experiments were performed in parallel in a 96-well plate and a set of microchannels representing various channel depths. **(D)** Transduction efficiency comparing a 96-well plate well with an ∼1.5 mm culture overlay and a 1.5 mm microchannel. Data points are presented as mean ±1 standard deviation (n = 2, 3). Significance was assessed using one-way ANOVA with Dunnett’s *post hoc* analysis (ns: p > 0.05; *: p < 0.05; **: p < 0.01; ***: p < 0.001; ****: p < 0.0001).

Analysing first the impact of different culture fluid overlays in the microchannels, the results in Figure 4A showed a statistically significant increase between the 1- and 0.2-mm channels when transduction is performed at constant MOI (p < 0.05, one-way ANOVA, Dunnett’s *post hoc* analysis). Figure 4B indicates that when the LVV concentration was kept constant at 6.3 × 10^5^ TU mL^-1^, the transduction efficiency correlated positively with the channel. In particular, there was a statistically significant decrease between the 1 and 0.2 mm microchannels when the LVV concentration was maintained constant (p < 0.05, one-way ANOVA, Dunnett’s *post hoc* analysis). Conversely, the percentage of GFP-positive cells correlated negatively with the channel depth when the transduction was performed at a constant MOI of 2. Consequently, this suggests that the total amount of particles loaded into a microchannel has a greater impact on the transduction efficiency than the concentration of LVV particles for the samples tested.

When evaluating a range of MOI values and comparing the microchannel results with the 96-well plates, a statistically significant increase in transduction efficiency in 0.2 mm channels was observed compared with 96-well plates, for all tested MOIs (0.5–4), and with p-values ranging from <0.001 to <0.05 (Figure 4C). The increase was approximately 2-fold in terms of the fraction of GFP-positive cells is observed across all MOI values tested relative to the 96-well plate instance. (MOI 0.5: 13% relative to 29%, representing an approximately 2-fold increase; MOI 1: 25% relative to 46%, 2-fold increase; MOI 2: 46% relative to 64%, 1.5-fold increase and MOI 4: 1.3-fold increase). In contrast, the 0.6 mm depth shows an increase in transduction efficiency only at high MOI values of 4. Similarly, the transduction performed at a fluid overlay of 1 mm shows relatively reduced transduction levels to the 96 well plate when tested at MOIs above 1 (p < 0.05, p < 0.01, one-way ANOVA, Dunnett’s *post hoc* analysis). This indicates that a significant increase in transduction efficiency across a range of MOIs can be obtained when constraining the interaction between lentiviral particles and target cell to a greater extent. By spatially constraining transduction events to depths equal to or smaller than 0.2 mm, a transduction efficiency of ≤20–30% (Shabram and Aguilar-Cordova, 2000) can be obtained from a 50% reduction in the number of particles. In the context of a cell-based functional titre quantification assay, this suggests an improvement in assay sensitivity of up to 50% across the 4 MOI values tested. This enables the detection of transduction efficiency and quantification of functional titre from samples with reduced LVV content (Supplementary Table S1).

To exclude that the microchannel data represent microfluidic artefacts, we performed LVV transductions in a 1.5 mm microchannel in parallel to 96-well plates with 50 µL volume per well; this volume corresponds to a fluid overlay of ∼1.5 mm. The results showed no statistically significant difference in transduction efficiency (p > 0.05, one-way ANOVA, Dunnett’s *post hoc* analysis) across a range of MOIs (0.5–4) (Figure 4D). The results from microchannels can therefore be directly compared with those from conventional well plates.

### 3.4 Evaluating assay linearity, reproducibility and effect of incubation time

**FIGURE 5:**
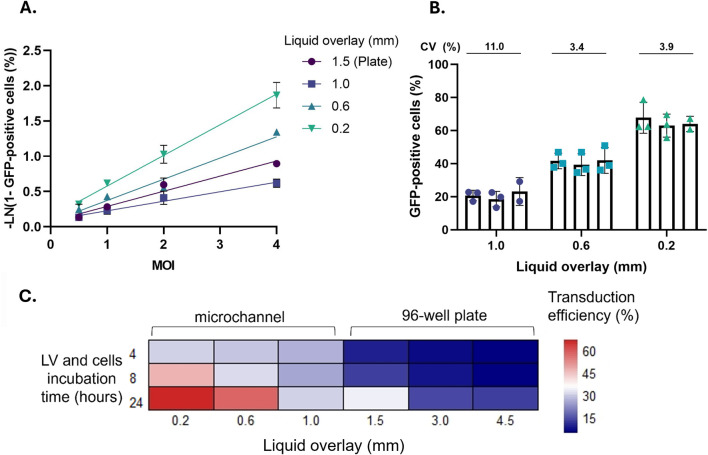
Assessment of linearity, reproducibility and incubation time of the microchannel assays. **(A)** Linearity evaluated using 4 MOI values, using the following channel depth: 1.5 mm (plate) and 1, 0.6 and 0.2 mm (microchannels). (1.5 mm: R^2^ = 0.9593; 1.0 mm: R^2^ = 0.8721; 0.6 mm: R^2^ = 0.9270; 0.2 mm: R^2^ = 0.9798). **(B)** Assay reproducibility evaluated across three experimental occasions for each channel depth (mm). Each experiment was performed in technical triplicates, i. e., for every channel depth three devices were evaluated in parallel. (CV: 1.0 mm: 11%; 0.6 mm: 3.4%; 0.2 mm: 3.9%). **(C)** Heatmap outlining the extent of transduction efficiency obtained across several incubation times between the LVV material and target cells. This is performed at a constant MOI of 1. All data is representative of the mean from triplicate measurements ±1 standard deviation. Significance was assessed using one-way ANOVA with Dunnett’s *post hoc* analysis (ns: p > 0.05; *: p < 0.05; **: p < 0.01; ***: p < 0.001; ****: p < 0.0001).

Assay linearity was evaluated across a range of MOI values for different culture fluid overlays (96-well plate and microchannels). The transduction efficiency was normalized by using Poisson regression and fitted with a simple linear model. The goodness of fit, indicated by the R^2^ value, for a 96-well plate (R^2^ = 0.9593) was comparable to the transductions in either 0.6 mm (R^2^ = 0.9270) and 0.2 mm (R^2^ = 0.9798) microchannels (Figure 5). A less favourable R^2^ was identified for the 1 mm channel (R^2^ = 0.8721), suggesting that potentially a spatial constraint to a greater extent is desirable in this case. Overall, the increase in normalised transduction efficiency followed a linear pattern across MOI values ranging from 0.5 to 4, indicating the suitability of microchannels for performing a functional titre quantification assay with a 0.2 mm culture fluid overlay representing the best performer.

The reproducibility of obtaining an increase in transduction efficiency across a series of culture fluid overlays was investigated by conducting 3 independent experimental occasions at constant MOI (Figure 5B). The 0.6 and 0.2 mm microchannels show a low coefficient of variation (CV) of 3.4% and 3.9%, respectively, indicating higher reproducibility. In contrast, the 1 mm channel had a CV of 11.0%. Though this CV is significantly higher than for other microchannels, the value still falls below the typically acceptable limit of 20% for cell-based assays (Wood et al., 2013). An increase in CV could be expected due to the inherent nature of cell-based assays, reflecting biological variation between experimental occasions performed on different days. In addition, the CV can be impacted by a series of external factors, including but not limited to operator error, imperfect microchannel microfabrication, bonding and operation or the impact of evaporation on the cell culture. However, this could represent an indication that by spatially constraining the interaction between target cells and viral particles to a greater extent, a potentially improved inter-assay precision can be achieved.

The effect of channel depth on transduction efficiency was evaluated for three different incubation times, representing strictly the variation of the ‘contact time’ between the LVV and target cells. As soon as the incubation time had elapsed, the LVV solution in the microchannel was replaced with fresh media. To allow for the expression of the gene of interest, the cells were cultured for at least an additional 48 h, followed by the quantification of transgene-positive cells by flow cytometry. For this experiment, the efficiency is evaluated across a wide range of culture fluid overlays in microchannels with depths of 0.2, 0.6 and 1 mm and in a 96-well plate with volumes of 50, 100 and 150 µL (corresponding to overlays of approximately 1.5, 3 and 4.5 mm, respectively) (Figure 5C). Transduction efficiency was higher in microchannels compared to a 96-well plate, across all incubation times. Notably, there appears to be an increase in transduction efficiency when comparing the 0.2 mm culture fluid overlay relative to the 96-well plate for a 4 h incubation. These results suggest the potential for a reduction in the overall time frame required to measure the transduction efficiency when quantifying the functional titre of an LVV sample. However, this would be applicable exclusively to the incubation time between LVV particles and target cells, while the time required to allow for gene expression remains constant.

### 3.5 Comparing assay sensitivity with a standard 96-well plate format

The results obtained thus far strongly suggest that minimising diffusion path lengths between adherent target cells and lentiviral particles increases transduction efficiency. We hypothesised that the enhanced proximity potentially also promoted a higher number of vector integrations per cell. The latter is crucial for analytical development as this would mean underestimating functional titre. While this effect can be corrected by accounting for the Poisson distribution (Shabram and Aguilar-Cordova, 2000), exploring this hypothesis was relevant to further characterise microchannel-based assays. In addition, clinical cell therapy programmes aim for fewer than 5 vector copies per cell, ideally <1 or 2, as recommended by regulatory guidelines, to avoid risks like insertional mutagenesis and cell population heterogeneity (Dan and Kang-Zheng, 2025).

The quantification of the vector copies per cell by qPCR is considered a more sensitive measure of transduction relative to determining the fraction of transgene-positive cells by flow cytometry (Zaninelli et al., 2024). Therefore, to properly assess the sensitivity of the microchannel format, both methods were used to measure transduction levels at very low MOIs (between 0.5 and 0.0625). With a constant cell seeding density of 7.5 × 10^4^ cells cm^-2^ in both microchannel and well plate, the cells had to be pooled from two consecutive transduction experiments to ensure sufficient cell material for both analytical methods. Consequently, the inter-assay variability introduced by performing the transductions on two separate occasions may confound the direct comparison of the two quantification methods.

**FIGURE 6:**
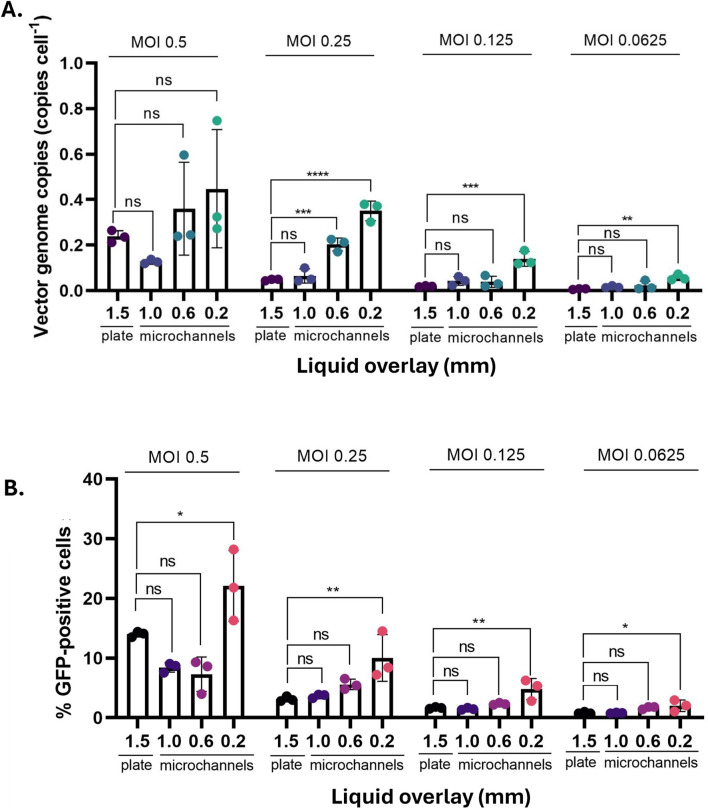
Identifying the impact of microchannel transductions on assay sensitivity compared with 96-well plates. **(A)** Quantification of vector genome copies per cell, measured by qPCR. **(B)** The fraction of GFP-positive cells determined by flow cytometry. All values are presented as mean of n = 3 with error bars indicating ±1 standard deviation of the mean. Significance was assessed using one-way ANOVA with Dunnett’s *post hoc* analysis (ns: p > 0.05; *: p < 0.05; **: p < 0.01; ***: p < 0.001; ****: p < 0.0001).

The number of vector integrations was quantified by measuring the vector packaging signal in extracted genomic DNA samples from microchannels at various MOI values, using a plate control as reference. As anticipated, due to the low MOI values employed in this experiment, the number of integrations per cell was very low, with all values below 1 copy per cell (Figure 6A). This finding aligned with the flow cytometry data (Figure 6B), with the highest value of 20% GFP-positive cells for the highest MOI in the thinnest culture fluid overlay. Together, these results indicate reduced transduction levels across the cell population due to the low MOIs tested.

The microchannels with a 0.2 mm overlay demonstrated a higher level of transduction efficiency and vector genome copies per cell, indicating enhanced sensitivity to detect minimal amounts of viral particles in the sample to be analysed, with MOIs as low as 0.0625. The 0.2 mm microchannel showed a statistically significant increase in transduction compared to the plate control, for both qPCR (p < 0.01, one-way ANOVA, Dunnett’s *post hoc* analysis) and flow cytometry (p < 0.05, one-way ANOVA, Dunnett’s *post hoc* analysis). This trend was observed across all MOIs when using a culture overlay of 0.2 mm (Figures 6A,B). Given that lower MOIs result in fewer particles targeting the adherent cell monolayer, a higher degree of spatial constraint may be necessary to achieve a statistically significant difference in transduction efficiency. Together, the results indicate that the 0.2 mm microchannel format has the highest sensitivity and the only case detecting MOIs as low as 0.0625.

## 4 Discussion

We have successfully developed and validated a novel microfluidic assay to quantify the functional titre of a lentiviral vector (LVV). Our devices, composed of chemically annealed PDMS on COC substrates (Figure 1), were confirmed to be sterile by autoclaving *via* a bioburden assay and a 14-day USP-compliant sterility test (Figure 2; Supplementary Figure S1). A systematic investigation of channel depths (0.2, 0.6, and 1.0 mm) confirmed that channel depth–and therefore mass transport - is a critical parameter of transduction performance (Ahmadi et al., 2023; Moore et al., 2019; Tran et al., 2017). At a constant multiplicity of infection (MOI), shallower channels significantly increased transduction efficiency (Figure 4A). Conversely, at constant LVV concentration, shallower channels significantly decreased transduction efficiency (Figure 4B) These effects were most pronounced for the 0.2 mm channel. When evaluated across a standard MOI range (0.5–4) and benchmarked against well plates, only the 0.2 mm channel provided a consistently higher transduction efficiency while maintaining a comparable reproducibility (Figure 4C) At low MOI values, the transduction efficiency increase amounted to approximately two-fold, pointing to a substantially higher sensitivity of the microfluidic assay.

To further validate the microfluidic approach, we evaluated the linearity of the assay for each channel depth, by analysing the normalised transduction efficiency (across MOIs from 0.5 to 4.) The 0.2 mm channel showed the strongest linearity (R^2^ = 0.9798), comparable to that of well plates (Figure 5A). The 0.2 mm channel furthermore had a coefficient of variation of (3.9%) from three technical replicates (three separately fabricated channels) within a single assay run (MOI = 1), highlighting the potential for improved inter-assay precision with greater spatial constraint (Figure 5B). As an additional operational advantage, the functional titre assay in 0.2 mm channels also provided a reduction in incubation time to just 4 h (Figure 5C).

Furthermore, the 0.2 mm channel exhibited the highest sensitivity, determined by two complimentary experiments: Firstly, it produced an approximate two-fold increase in transgene-positive cells across the standard MOI range (0.5–4) (Figure 4C). Secondly, the 0.2 mm channel was the only configuration–well plates included–to reliably detect transduction at very low MOIs (down to 0.0625) (Figure 6). This enhanced low-level detection was corroborated by qPCR analysis of vector genome integration, which confirmed the 0.2 mm channel was uniquely capable of detecting a signal at this limit. Collectively, these findings established the 0.2 mm microchannel as a new performance benchmark, delivering superior sensitivity alongside assay reproducibility and linearity.

The increased sensitivity is particularly attractive for in-process testing during process development steps, where detecting low levels of LVV in samples with small amounts of viral vector content is essential. An enhanced assay sensitivity is also advantageous in cell line development, for the generation of stable LVV producers, as it enables sequential high throughput screening steps at lower titres, typical for operation at small scale.

Our study further highlighted an opportunity for assay standardisation. The 1.5 mm microchannel and the 50 µL well plate performed identically (Figure 4D). This result extends beyond serving as a control: it not only confirms that the substantial variations observed in TE across different channel depths are not a microfluidic artefact, nor simply validates that comparable results can be achieved when identical workflows are used in both microfluidic or well plate format. Rather, it highlights that a microfluidic approach enables controlled and systematic variation of the culture environment to identify an improved assay standard.

That an optimal culture fluid overlay thickness lies within a few tenths of a millimetre is consistent with both our findings and historical virology studies. Early investigations of host-virus, such as plaque assays, employed very thin fluid overlays; published protocols for plaque assays employed fluid overlays on the order of a 10th of a millimetre (Dulbecco and Vogt, 1954) to minimise titre underestimation (Shabram and Aguilar-Cordova, 2000). For larger overlays, previous reports pointed out that the effective diffusion distance for LVV particles at 37 °C is approximately 0.6 mm before substantial decrease in particle half-life occurs (Chuck and Palsson, 1996). This aligns with our results: we observed higher transduction efficiency and better reproducibility in 0.2 mm channels compared with 0.6 mm. Even thicker culture fluid overlays, such as in well plates, further exacerbate titre underestimation by reducing the likelihood of productive virus-cell interactions (Andreadis et al., 2000). There is also likely a lower limit. Tran et al. (2017) reported a decline in transduction efficiency below approximately 0.1 mm, i.e., when the volume dropped below 3 μL at a given MOI. Our own results indicate challenges of maintaining cell viability in very thin microchannels for longer than 72 h (Figure 3). Reduced overlay heights limits nutrient availability for the cells, creating a practical limit for very thin microchannels.

To fully standardise the assay, additional factors must be considered. In line with our findings (Figure 5C), variations in the incubation time between LVV particles and cells substantially impact TE, leading to discrepancies when determining functional titre. Beyond this, cell-based functional assays inherently reflect the biological variability of cell cultures (Lin-Gibson et al., 2016; Riss et al., 2021). Variability is further compounded by a lack of standardisation across LVV titration methodologies. Differences in target cell type, incubation duration, MOI selection, and gating strategies used during downstream analysis can all strongly affect the resulting functional titre. Together, all these effects have historically limited direct comparisons across studies conducted under different experimental conditions (Merten et al., 2016; Nyberg-Hoffman et al., 1997; Perry and Rayat, 2021; Sinn et al., 2005).

Current regulatory guidance similarly states that infectious titre determination should be product-specific and reflective of the mechanism of action, without prescribing a standardised assay format or experimental conditions (Department of Health and Human Services, 2011). While this regulatory framework allows for flexibility, it concomitantly perpetuates methodological heterogeneity and limits inter-laboratory comparability. Consequently, the need for developing international reference standards for viral vectors has been widely acknowledged, with an LVV reference material made recently available (Manceur et al., 2025).

Further development of this approach could focus on workflow automation. The current microfluidic devices relied on extensive manual pipetting, and automating liquid handling would remove operator-dependent variability. In addition, replacing flow cytometry with fluorescence microscopy-based quantification of adherent cells would streamline readout and enable real-time monitoring (Labisch et al., 2021). Coupling this with AI-driven image analysis could further accelerate data processing and reduce assay burden (Han et al., 2024). Together, these advances would further enhance reproducibility and standardisation, thereby accelerating LVV development and improving inter-laboratory comparability.

In summary, we have successfully demonstrated that microfluidics can be leveraged to deliver a reliable analytical assay for functional titre quantification. By systematically characterising the thickness of the culture fluid overlay, i.e., the microchannel depth, we have identified this parameter as a key design variable for achieving a better-defined, more sensitive analytical system. The 0.2 mm channel provides linearity and reproducibility comparable to 96-well plates, whilst reducing incubation periods. Critically, the enhanced analytical sensitivity (limit of detection down to MOI 0.0625) enables robust detection of very low viral particle-to-cell ratios at which well plates fail. Thus, the microfluidic offers a more controlled and engineered microenvironment than conventional well plates, creating an opportunity to shift the functional titre assay from a protocol-based procedure to a device-based analytical standard.

## Data Availability

The raw data supporting the conclusions of this article will be made available by the authors, without undue reservation.
