# Supplementary material for: Rethinking viral vector quantification: a microfluidic approach to standardised functional titre assays
Source: Front Bioeng Biotechnol. 2026 Apr 13;14:1720882. doi: 10.3389/fbioe.2026.1720882 (PMC13111257; doi:10.3389/fbioe.2026.1720882)
Supplement: Supplementary file 1 [file DataSheet1.pdf]

Supplementary Material

1 Supplementary Figures and Tables

1.1 Supplementary Figures

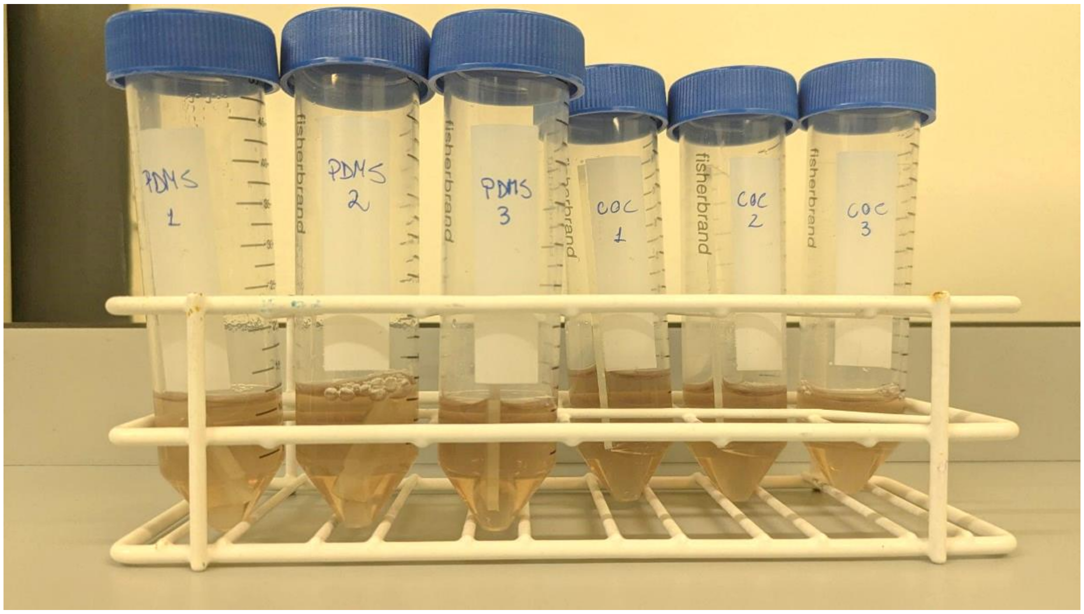

Figure S1. Visual observation of cultures obtained after 14 days static incubation of one part of poly-dimethyl-siloxane (PDMS) and cyclo-olefin co-polymer (COC) each in Fluid Thioglycolate Medium (FTM, Fisher Scientific, UK) at 35°C according to United States Pharmacopeia (USP) 71 guidelines. Visual observation indicated no contamination growth under these conditions.

1.2 Supplementary Tables

Table S1. Calculations for functional titre quantification (TU mL^{-1}) derived from the transduction efficiency obtained from either a 96-well plate or a microchannels representing 1, 0.6 and 0.2 culture liquid overlay. The calculations are performed by adding the same amount of virus to each assay format, equivalent of a multiplicity of infection (MOI) of 0.5.

Assay format	Transduction volume (mL)	% GFP positive cells	Dilution factor	No of cells at transduction (TU mL^{-1}, x 10^{4})	Average (TU mL^{-1}, x 10^{9})	Standard deviation (TU mL^{-1}, x 10^{9})	CV%
Plate	0.05	0.12	30000	5.50	4.15	0.19	4.80
		0.125					
		0.132					
Microchannel (1 mm)	0.042	0.123	25500		4.22	0.38	9.00
		0.139					
		0.117					
Microchannel (0.6 mm)	0.0252	0.275	15000		8.02	1.39	17.32
		0.215					
		0.16					
Microchannel (0.2 mm)	0.0084	0.289	5000		8.97	0.43	4.77
		0.268					
		0.265					
